# Supplementary material for: Vision and vocal communication guide three-dimensional spatial coordination of zebra finches during wind-tunnel flights
Source: Nat Ecol Evol. 2022 Jun 30;6(8):1221–30. doi: 10.1038/s41559-022-01800-4 (PMC9349042; doi:10.1038/s41559-022-01800-4)
Supplement: Supplementary file 1 — Supplementary Tables 1 and 2, and captions of Supplementary Videos 1–5. [file 41559_2022_1800_MOESM1_ESM.pdf]

nature portfolio

https://doi.org/10.1038/s41559-022-01800-4

Supplementary information

Vision and vocal communication guide three-dimensional spatial coordination of zebra finches during wind-tunnel flights

In the format provided by the authors and unedited

Supplementary Information for

Vision and vocal communication guide 3D spatial coordination of zebra finches during wind-tunnel flights

Fabian Arnold, Michael S. Staniszewski, Lisa Pelzl, Claudia Ramenda, Manfred Gahr, Susanne Hoffmann

Correspondence to: shoffmann@orn.mpg.de

This file includes:

Supplementary Text

Supplementary References

Supplementary Tables 1 and 2

Captions for Supplementary Movies 1 to 5

Supplementary Text

Birds' spatial preference in the flight section

Although the birds occupied the entire width of the flight section, they generally preferred to fly left to its horizontal center. In contrast to the right side of the flight section, the left-side walls were transparent, and the birds' home aviary was visible through the left-side walls. Both the difference in wall transparency and the view towards the home aviary could explain the bird's tendency to fly on the left side in the flight section.

Position change frequencies during solo flight

To test for the dependency of the birds' behavior on the social context, we reran the experiment and interspersed flocking flight sessions with flight sessions in which each bird flew solo (four sessions per bird) in the wind tunnel's flight section. While all birds maintained their preferred flight area in the flight section when flying solo (Extended Data Fig. 3), they showed an even more dynamic flight behavior than during flocking flight (Extended Data Fig. 4). The frequency of rhythmic horizontal position changes during solo flight (group mean ± STD: 0.42 ± 0.09 Hz, (i.e., cycles per second), $n = 24$) was significantly higher (Linear mixed model (LMM), estimates ± SE: 0.07 ± 0.02, $p = 0.002$, $t = 3.33$) than in the flocking flight condition (group mean ± STD: 0.35 ± 0.12 Hz, $n = 24$). And the frequency of rhythmic vertical position changes during solo flight (group mean ± STD: 0.19 ± 0.21 Hz, $n = 24$) was also significantly higher (LMM, estimates ± SE: 0.14 ± 0.04, $p = 0.002$, $t = 3.27$) than in the flocking flight condition (group mean ± STD: 0.05 ± 0.09 Hz, $n = 24$). A large variability in flight position was also observed in homing pigeons (*Columba livia*) when flying solo, and has been suggested to aid in predator avoidance when flocking is not possible [38].

Alignment of movements between flock members

When flying in the wind tunnel, the birds' main direction of motion was forced by the air flow and the main flight direction was therefore artificially aligned amongst all flock members throughout the flight session. Because the birds moved against the air flow, their position in wind direction roughly remained constant, which prevented the camera system to capture any alignment of motion. Significant positional changes in wind direction were only rarely performed. Except for alignment in the main flight direction, we did not expect the zebra finches to align movement directions among all members of the flock. A correlational analysis of movement directions in the footage of *Camera 1* revealed that for all possible pairwise combinations of birds, the degree of coordination of movement directions among two individuals constantly alternated between periods of alignment (correlation coefficient > 0) and periods of misalignment (correlation coefficient < 0). Synchronous with events of alignment in one or more pairs of birds, birds of other pairwise combinations moved in directions opposite to each other (Fig. 1b and c; Extended Data Fig. 5), which caused the degree of coordination averaged over all bird pairings to be close to zero throughout all four flight sessions (Extended Data Fig. 5). However, in each of the four flight sessions, at least one short time period was detected, during which movement directions were significantly aligned among the majority of individuals in the flock (i.e., mean correlation coefficient above 0.3, mean P value below 0.05; Extended Data Fig. 5). We did not detect specific behaviors correlated with these brief events of significant alignment and therefore suggest that they occurred by chance. To determine whether the birds are able to intentionally align their movement directions, additional experiments are necessary during which birds in the flock are forced to change their movement directions collectively.

Head turning behavior during solo flight

It is important to note that head turning behavior during horizontal position changes was independent of the social context, and was observed in both flocking flight and solo flight sessions (see Supplementary Video 3 for examples of horizontal position changes during solo flight). Neither the mean angle of absolute position change, nor the mean absolute turning angle of the birds' heads, nor the mean delay between initiation of head turn and initiation of position change differed significantly (LME, $p > 0.4$, $t < 0.8$ for all measurements, $n = 60$ per condition) between conditions. This suggests that while flying, zebra finches use visual cues to avoid collisions with both moving flock members and static obstacles, such as the flight section's walls.

Spatial arrangement of the flock at the initiation of upwards directed movements

In all three dimensions, horizontal, vertical and wind direction, the relative distance between a bird that was moving upwards without calling and its flock mates ($n = 2637$ for azimuth and elevation, and $n = 2284$ for wind direction) at the time of movement initiation was significantly (LMM, estimates ± SE: 80.5 ± 19.9, $p < 0.001$, $t = 4.04$, estimates ± SE: -127.2 ± 12.5, $p < 0.001$, $t = 10.17$, and estimates ± SE: 105.5 ± 37.9, $p = 0.005$, $t = 2.78$, respectively) different from the relative distance between a bird that was moving upwards after emitting a call and its flock mates ($n = 230$ for azimuth and elevation, and $n = 202$ for wind direction). To exclude the possibility that call emission is solely triggered by the flock arrangement, we counted events within the four fully tracked flight sessions during which the median position of flock mates was further left, further up and further downwind to the position of a focal bird. The average ratio between the number of Stack call emissions and the number of specified flock arrangement events was 10.25:135 (2:67, 7:112, 14:153, 18:208). This means that on average only 1 in 14 of these particular flock arrangement events could have been associated with a Stack call, which opposes the hypothesis that a bird calls whenever its flock mates are in a particular position relative to this bird.

Effect of wind noise on vocal communication

The peak frequencies of the wind noise measured at a wind speed of 10 m/sec in the flight section were below 1000 Hz, and the high-frequency cut off at half amplitude was below 2000 Hz (Extended Data Fig. 9a). In the frequency range between 2000 and 5000 Hz, the mean amplitude of Stack calls measured at a distance of 50 cm in front of zebra finches perched in the calm flight section exceeded by up to 10 dB the mean of wind noise amplitudes measured at nine different spatial positions in the flight section at a wind speed of 10 m/sec (Extended Data Fig. 9a). Therefore, we expect the masking effect the wind noise in the flight section had on Stack calls emitted during flight at a wind speed of 10 m/s to be moderate, and comparable to the effect wind noise has on calls of zebra finches flying at a speed of 10 m/sec in free space.

Supplementary References

38 Garde, B. et al. Fine-scale changes in speed and altitude suggest protean movements in homing pigeon flights. Royal Society open science 8, 210130, doi:10.1098/rsos.210130 (2021).

	Mean horizontal distance [% of maximum ± SEM]	Mean vertical distance [% of maximum ±SEM]	Mean distance in wind direction [% of maximum ±SEM]
**Session #2**	30.3 ± 0.2	31.7 ± 0.2	36.0 ± 0.3
**Session #5**	34.7 ± 0.2	30.9 ± 0.2	35.8 ± 0.2
**Session #8**	33.7 ± 0.2	31.5 ± 0.1	38.0 ± 0.2
**Session #13**	30.8 ± 0.1	27.7 ± 0.1	34.8 ± 0.2

Supplementary Table 1 | Spatial distances between birds. Mean and standard error of the mean (SEM) of spatial distances between two birds measured throughout four flight sessions for all possible bird pairings. Each distance measurement was normalized to the maximum distance detected for the particular pairing, dimension, and flight session.

Flight Condition	# of sessions performed	# of sessions analyzed				
		Flight behavior	Head turns	Call-related movements	Call rates	Collisions
**Flock**	13	4	1	12	13	13
**Solo**	24 (4 per bird)	24 (4 per bird)	6 (1 per bird)	-	19	-
**0.2 Lux**	10	-	-	-	10	10
**20 Lux**	10	-	-	-	10	10
**LP noise**	10	-	-	-	10	10
**BP noise**	10	-	-	-	10	10

Supplementary Table 2 | Overview of sample sizes. The number of recorded and analyzed flight sessions is shown for each flight condition performed and each behavioral parameter investigated. Please note that the microphone signal quality of bird *Black* was bad throughout all flight sessions, and the microphone signal of bird *Green* was bad throughout one solo flight session. Therefore, microphone data from these sessions and birds were excluded from the analysis of call rates. LP noise: low-pass filtered background noise (control), BP noise: band-pass filtered background noise (masker).

Supplementary Movie 1 | Flight behavior of a flock of zebra finches in the wind tunnel's flight section. The video clip recorded by *Camera 1* shows the flight behavior in the horizontal/vertical plane of six zebra finches during flocking flight at 200 Lux in the wind tunnel's flight section. Please note the frequent horizontal position changes performed by individual birds.

Supplementary Movie 2 | Head turning during horizontal position changes in flocking flight. The video clip recorded by *Camera 2* shows the flight behavior in the horizontal/wind direction plane of six zebra finches during flocking flight in the flight section. After seven seconds, the video is slowed down ten times for better visibility of head turns during horizontal position changes.

Supplementary Movie 3 | Head turning during horizontal position changes in solo flight. The video clip recorded by *Camera 2* shows two horizontal position changes performed by bird *Orange* during solo flight. At the beginning of the clip, the video is displayed at normal speed and subsequently the same sequence is displayed slowed down ten times for better visibility of the bird's behavior.

Supplementary Movie 4 | Call-accompanied upwards directed position change during flocking flight. The video clip recorded by *Camera 1* shows a call-accompanied upwards directed position change performed by bird *Green* during flocking flight. At the beginning of the clip, the video is displayed at normal speed, and subsequently the same sequence is displayed slowed down ten times for better visibility of the bird's behavior. Bird *Green* is marked by a red circle at the onset of the call it emitted before moving upwards. The audio signal of bird *Green*'s microphone transmitter has been added to the video clip as sound trace.

Supplementary Movie 5 | Collision between birds during flight in the presence of masking noise. The video clip with footage synchronously recorded by *Camera 1* (left screen) and *Camera 2* (right screen) shows two birds colliding during flocking flight from two different viewing directions. At the beginning and the end of the clip, the video is displayed at normal speed, but slowed down ten times during the collision, which can be seen in the middle of each screen.
